# Association of the EGF-TM7 receptor CD97 expression with FLT3-ITD in acute myeloid leukemia

**DOI:** 10.18632/oncotarget.5661

**Published:** 2015-10-07

**Authors:** Manja Wobus, Martin Bornhäuser, Angela Jacobi, Martin Kräter, Oliver Otto, Claudia Ortlepp, Jochen Guck, Gerhard Ehninger, Christian Thiede, Uta Oelschlägel

**Affiliations:** ^1^ Division of Hematology, Oncology and Stem Cell Transplantation, Department of Medicine I, University Hospital Carl Gustav Carus, Technische Universität, Dresden, Germany; ^2^ Biotechnology Center, Technische Universität Dresden, Dresden, Germany; ^3^ German Consortium for Translational Cancer Research (DKTK), Heidelberg, Germany

**Keywords:** AML, CD97, FLT3-ITD, bone marrow microenvironment

## Abstract

Internal tandem duplications within the juxtamembrane region of the FMS-like tyrosine kinase receptor FLT3 (FLT3-ITD) represents one of the most common mutations in patients with acute myeloid leukemia (AML) which results in constitutive aberrant activation, increased proliferation of leukemic progenitors and is associated with an aggressive clinical phenotype. The expression of CD97, an EGF-TM7 receptor, has been linked to invasive behavior in thyroid and colorectal cancer. Here, we have investigated the association of CD97 with FLT3-ITD and its functional consequences in AML.

Higher CD97 expression levels have been detected in 208 out of 385 primary AML samples. This was accompanied by a significantly increased bone marrow blast count as well as by mutations in the *FLT3* gene. FLT3-ITD expressing cell lines as MV4-11 and MOLM-13 revealed significantly higher CD97 levels than FLT3 wildtype EOL-1, OCI-AML3 and HL-60 cells which were clearly decreased by the tyrosine kinase inhibitors PKC412 and SU5614. CD97 knock down by short hairpin RNA in MV4-11 cells resulted in inhibited trans-well migration towards fetal calf serum (FCS) and lysophosphatidic acid (LPA) being at least in part Rho-A dependent. Moreover, knock down of CD97 led to an altered mechanical phenotype, reduced adhesion to a stromal layer and lower wildtype FLT3 expression.

Our results, thus, constitute the first evidence for the functional relevance of CD97 expression in FLT3-ITD AML cells rendering it a potential new theragnostic target.

## INTRODUCTION

Acute myeloid leukemia (AML) is a heterogeneous group of diseases and the most frequent leukemia subtype in adult patients. It is characterized by an increase in the number of malignant myeloid progenitors in the bone marrow and an arrest of their maturation, frequently resulting in hematopoietic insufficiency (granulocytopenia, thrombocytopenia, or anemia), with or without leukocytosis [1]. Mesenchymal stromal cells (MSCs) have been described as a major component of the bone marrow microenvironment. In analogy to their support of early hematopoietic stem and progenitor cells (HSPCs) they have been demonstrated to induce adhesion-mediated chemoresistance of clonogenic leukemic cells [2]. Several chemokines, cytokines and adhesion molecules are involved in the crosstalk between HSPCs or leukemic cells and their stromal microenvironment. Moreover, cell mechanics could be related to dynamic interactions between different cell types. The ability for migration through tissue or endothelial layers as well as for circulation through the microvasculature depends on cell stiffness. The more deformable, the easier and more effective these processes should be facilitated [3, 4]. Chemotherapy was shown to increase leukemia cell stiffness which occurred before caspase activation and peaked after completion of cell death [5].

The internal tandem duplication of the FLT3 receptor (FLT3-ITD) is the most common mutation in AML with a frequency of about 25%. These mutations are correlated with a poor prognosis and a high amount of peripheral blood blasts [6]. FLT3 mutations occur more frequently in patients with normal karyotype and have a higher recurrence rate after conventional chemotherapy [7]. Activation of FLT3 by mutation results in autophosphorylation as well as phosphorylation of a number of proteins, either directly or indirectly.

CD97 is the founding member of the EGF-TM7 molecule family, a subgroup of adhesion G-protein coupled receptors. It is expressed on the surface of lymphocytes, monocytes, macrophages, dendritic cells, granulocytes and smooth muscle cells [8]. CD97 upregulation can be detected during activation of lymphocytes. The molecule has been implicated in cell adhesion and migration through interactions with cell surface proteins and components of the extracellular matrix (ECM) [9]. CD97 has four known ligands: CD55, a negative regulator of the complement cascade [10], chondroitin sulfate, a component of the ECM [11], CD90 (Thy-1) [12] and the integrin α5β1 [13]. An association with integrins is important for the mediation of invasion, migration and angiogenesis in solid cancer [13]. In undifferentiated anaplastic thyroid carcinoma high CD97 expression was found to be associated with metastatic lesions [14]. Colorectal carcinomas with increased CD97 staining in scattered tumor cells showed a poorer clinical stage as well as increased lymph vessel invasion compared to cases with uniform CD97 expression [15]. So far, almost nothing is known about CD97 expression and biological function in normal and malignant human HSPCs.

In the present study, we demonstrate for the first time an increased CD97 expression in AML cells preferentially carrying FLT3-ITD. Adhesion, deformability and migration of FLT3-ITD leukemia cells are all dependent on CD97 expression.

## RESULTS

### CD97 expression in AML patient samples correlates with *FLT3* and *NPM1* mutation

**Figure 1:**
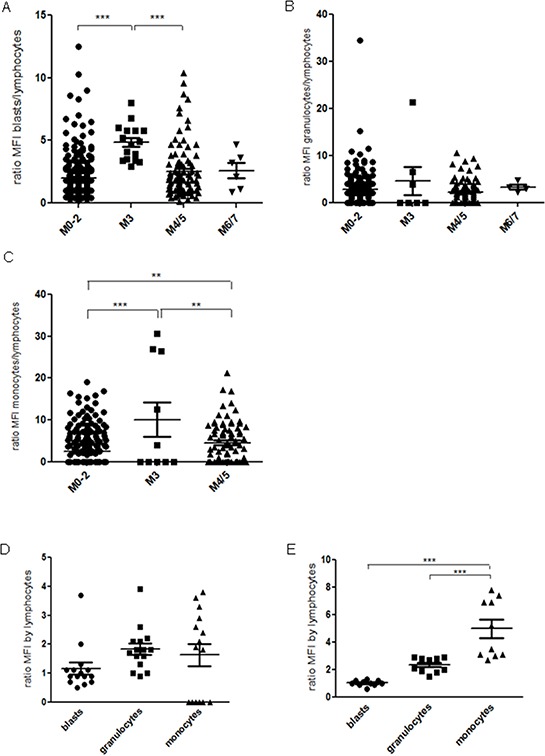
Bone marrow samples of 385 de novo AML patients were investigated by flow cytometry CD97 expression is shown as a ratio of mean fluorescence intensity (MFI) of **A.** blasts, **B.** granulocytes, or **C.** monocytes in relation to the MFI of lymphocytes in each sample according to the AML classification as well as for **D.** MDS and **E.** healthy control samples. The line indicates the mean. *p* < 0.01 (**), *p* < 0.001 (***).

Flow cytometric analysis was performed in a patient sample collective. We detected significantly higher CD97 expression levels (mean fluorescence intensity, MFI) in 208 out of 385 samples compared to bone marrow blasts from healthy donors (*n* = 10) and MDS patients (*n* = 15). In detail, CD97 expression could be observed in 131 out of 272 cases with M0-2, all of 16 cases with M3, 57 out of 91 patients with M4/5 and 4 out of 6 M6/7 cases, respectively (Figure 1). Of note, higher CD97 expression was accompanied by a significantly higher bone marrow blast count (75% vs. 53%, *p* < 0.001) and a lower Hb (5.9 vs. 6.5, *p* = 0.02). Interestingly, elevated CD97 expression in blasts was associated with mutations in *NPM1* (37% vs. 15%, *p* < 0.0001) and *FLT3* genes (38% vs. 10%, *p* < 0.0001) as well as lower CD34 expression (52% vs. 78%, *p* < 0.0001) (Table 1).

**Table 1:** Case distribution according to the AML FAB classification and phenotypical analysis by flow cytometry with respect to CD97 expression

	CD97 neg		CD97 pos		
	number	percent	number	percent	*P* value
patients	177		208		
AML M0-2	141	79	131	63	0.0005
AML M3	0	0	16	8	<0.001
AML M4/5	34	19	57	27	0.0709
AML M6/7	2	1	4	2	ns
					
CD34+	138/177	78	109/208	52	<0.001
CD13+	153/165	93	179/192	93	ns
CD33+	159/177	94	193/198	97	0.0756
CD117+	166/171	97	180/199	90	0.0107
HLA-DR+	149/165	90	166/192	86	ns
CD56+	62/178	35	62/208	30	ns
CD2+ and/or CD7+	21/178	12	21/208	10	ns
7.1+	11	6	15	7	ns
*mNPM*	25/167	15	71/193	37	<0.001
*mFLT3*	17/164	10	73/194	38	<0.001
good risk genetics	11/164	7	33/194	17	0.0035
*mNPM* only	16/167	10	32/193	17	0.0619

Abbreviations. ns: not significant; m: mutated; neg: negative; pos: positive

A significant higher percentage of M3 cases displayed elevated CD97 expression in leukemic cells than samples of M0-2 or M4/5 (Figure 1A). Whereas no significant differences between the AML subgroups could be detected in granulocytes (Figure 1B), residual monocytic cells displayed significantly different CD97 expression levels (Figure 1C). Although CD97 expression tended to be higher in granulocytes and monocytes of MDS samples, no significant differences could be detected in comparison to the expression in blasts (Figure 1D). Healthy bone marrow samples displayed significantly higher CD97 expression in granulocytes and monocytes than blasts (Figure 1E).

From the primary patient sample data, we found the correlation between higher CD97 expression and FLT3-ITD the most clinically relevant. Therefore, further *in vitro* investigations were focused on this association.

### CD97 expression is higher in FLT3-ITD AML cells *in vitro* and can be inhibited by tyrosine kinase inhibitors

**Figure 2:**
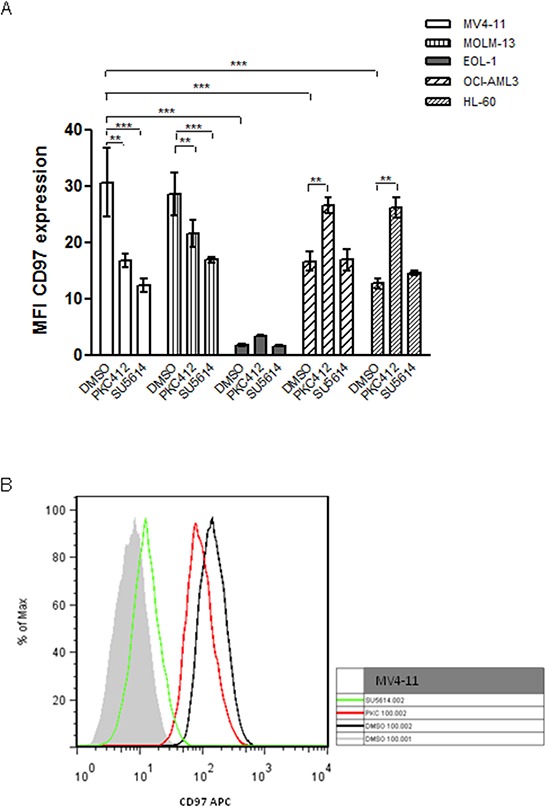
FACS analysis of CD97 expression in AML cell lines with different FLT3 mutation state **A.** CD97 expression levels in FLT3-ITD carrying MV4-11 and MOLM-13 cells were significantly higher compared to EOL-1, OCI-AML3 and HL-60 cells. Treatment of MV4-11 and MOLM-13 cells with 0.5 μM of the tyrosine kinase inhibitor PKC412 or 0.1 μM SU5614 significantly decreased the CD97 expression whereas the low CD97 expression levels in EOL-1 cells were not affected by these inhibitors. CD97 levels in OCI-AML3 and HL-60 cells were even increased by PKC412 treatment. Data is represented as mean ± SEM of three independent experiments, *p* < 0.05 (*); *p* < 0.01 (**); *p* < 0.001 (***). **B.** One representative measurement for MV4-11 cells is displayed. Filled grey: isotype control; black: CD97-APC in DMSO treated control cells; red: CD97-APC in PKC412 treated cells; green: CD97-APC in SU5614 treated cells.

The expression of CD97 in leukemic cell lines with different FLT3 status was investigated by flow cytometry. Interestingly, MV4-11 and MOLM-13 cells which carry FLT3-ITD displayed significantly higher CD97 levels (MFI 30.6 and 28.8, respectively) compared to FLT3 wildtyp EOL-1 and HL-60 cells (MFI 1.7 and 12.6, respectively; Figure 2A). OCI-AML3 cells which are FLT3 wildtype but mutated in the NPM1 gene revealed median CD97 expression levels (MFI 16.6; Figure 2A). These data were confirmed at the mRNA level by real-time PCR (not shown).

To further investigate the effect of endogenous FLT3-ITD on CD97 expression the small molecule inhibitors PKC412 and SU5614 were used to block FLT3 action. Short-term treatment of the cell lines with sublethal doses (0.5 μM PKC412; 0.1 μM SU5614) of the tyrosine kinase inhibitors resulted in significantly decreased CD97 expression in MV4-11 and MOLM-13 cells (MFI 30.6 or 28.6 in DMSO treated control cells, MFI 16.7 or 21.5 in PKC412 treated cells, MFI 12.4 or 16.8 in SU5614 treated cells) but no changes in EOL-1 cells after 12 h (Figure 2A, 2B). In OCI-AML3 and HL-60 cells PKC412 caused a significantly increased CD97 expression (Figure 2A) underlying the additional regulation of other tyrosine kinases by this inhibitor. The viability of cells was not affected as tested by trypan blue exclusion (not shown).

### CD97 knock down in MV4-11 cells affects FLT3 expression

**Figure 3:**
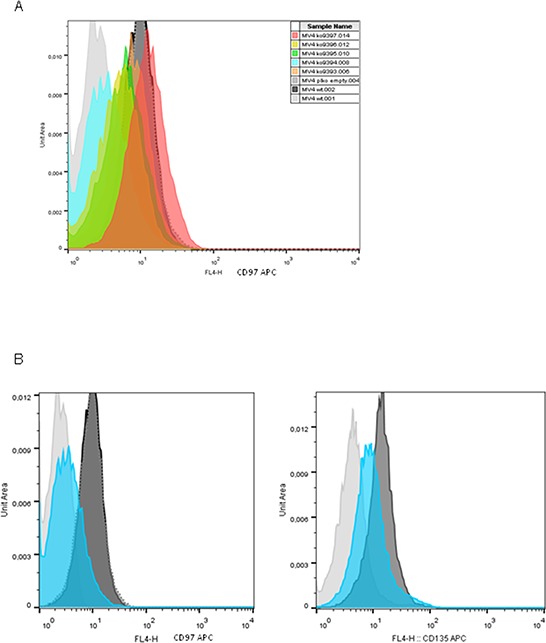
CD97 expression in MV4-11 cells can be knocke down by shRNA which also affects FLT3 expression **A.** Different shRNAs down-regulated CD97 expression with various efficiency as measured by flow cytometry using anti-CD97-APC antibodies in comparison to the isotype control IgG-APC (filled grey). One representative measurement is shown. **B.** The most effective shRNA targeting CD97 also down-regulated wildtype FLT3 (CD135) expression as shown by flow cytometry. One representative measurement is presented for CD97-APC (left) and CD135-APC (right).

To investigate potential physiological functions associated with CD97 in leukemia, endogenous expression was knocked down using ectopic short hairpin RNA (shRNA) in MV4-11 cells. As shown in Figure 3A, the five tested shRNAs regulated the CD97 expression with different efficiency. The empty plko.1 vector served as control. Since shRNA 9394 regulated CD97 almost to the control levels, we performed further experiments with MV4-11 cells transduced with this clone in comparison to the empty plko.1 vector. Interestingly, CD97 knock down inhibited also the expression of FLT3 (CD135) as measured by flow cytometry (Figure 3B) underlying the association of both receptors. The CD97 knock down in MV4-11 cells had no influence on metabolic activity and apoptosis as measured by MTT assay and annexin-V/propidium iodide staining (not shown).

### CD97 knock down in MV4-11 cells modulates migration, deformability and adhesion potential

**Figure 4:**
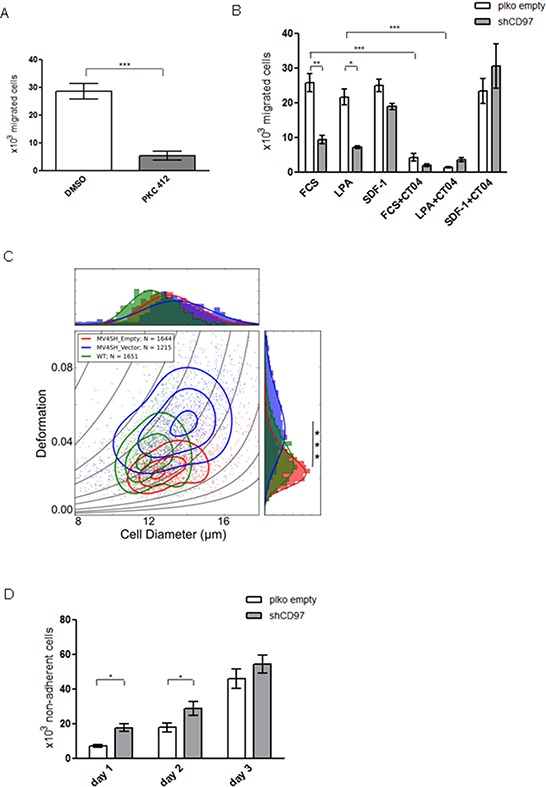
The migration, deformability and adhesion capacity of MV4-11 cells is modulated by CD97 knock down **A.** Trans-well migration of MV4-11 cells towards FCS in a Boyden chamber assay was significantly inhibited after treatment with PKC412. Data is represented as mean ± SEM of three independent experiments, *p* < 0.001 (***). **B.** Migration capacity of CD97 knock down MV4-11 cells is significantly decreased in comparison to control cells towards FCS and LPA but not affected towards SDF-1. Pre-treatment of the cells with the Rho inhibitor CT04 resulted in decreased migration potency of both cell types towards FCS and LPA whereas migration towards SDF-1 was not impaired. Data is represented as mean ± SEM of three independent experiments, *p* < 0.05 (*); *p* < 0.01 (**); *p* < 0.001 (***). **C.** RT-DC on MV4-11 cells. Mechanical properties (wildtype - green, empty vector - red, CD97 down-regulated - blue) were determined in separate experiments and revealed distinct populations in size and deformation (indicated by 1d projection at top and right). Data acquisition was carried out in a 20 μm x 20 μm channel at a flow rate of 0.04 μl/s and summarize *n* = 2,688 (wildtype), *n* = 2009 (empty vector) and *n* = 2404 (CD97) single cell measurements. Curved lines are isoelasticity lines and contour lines represent 90%, 50% and 20% of maximum intensity, respectively. Statistical significance between wildtype and CD97 knock down (***p < 0.00001). **D.** Adhesion of CD97 knock down and control MV4-11 cells to a MSC monolayer was compared at 1, 2 and 3 days by counting the cells in the supernatant. Data is represented as mean ± SEM of three independent experiments, *p* < 0.05 (*).

PKC412 treatment of MV4-11 cells was shown to suppress FLT3 and CD97 expression levels. To investigate a potential functional consequence, we tested *in vitro* trans-well migration capacity of the cells towards FCS. As shown in Figure 4A, PKC412 treated cells displayed a significantly reduced migration rate compared to the control suggesting an influence of FLT3 and probably CD97 on the leukemic cell migration potential.

Next, we analyzed the impact of CD97 expression on the migration capacity of MV4-11 cells in more detail. Induction of spontaneous migration by 10% FCS revealed a significantly lower migration rate of CD97 knock down MV4-11 cells compared to those carrying the empty vector (Figure 4B). Lysophosphatidic acid (LPA) is a major chemoattractant found in serum which was already demonstrated to impact CD97-dependent migration capacity of prostate cancer cells [16]. Therefore, different LPA concentrations (1.0/0.1/0.01/0.001 μM) were applied to test chemoattractivity of MV4-11 cells. A concentration of 0.1 μM was found to be most potent (not shown) and therefore used in the following experiments. Migration of CD97 knock down MV4-11 cells was again significantly reduced in comparison to cells containing the empty vector (Figure 4B). In contrast, the SDF-1/CXCR4 axis which is important for migration and homing of both normal HSPCs and leukemic cells is not involved in CD97-mediated migration processes since there were no significant differences in the percentage of migrated cells towards recombinant SDF-1 (Figure 4B). Pre-treatment of MV4-11 cells with the cell permeable Rho inhibitor CT04 suppressed migration towards FCS and LPA of both control and CD97 knock down cells (Figure 4B), suggesting that most of the CD97-dependent migration was Rho-A dependent.

Also changes in the cytoskeletal arrangement, manifested in the overall deformability of cells, have been implicated in their ability to migrate [4] which can be detected by RT-DC and quantified by the degree of deformation. Comparing the untransduced wildtype (green), the empty vector control (red) and the CD97 knock down MV4-11 cells (blue) revealed three distinct populations which are highlighted by contour lines of 90%, 50% and 20% of the maximum density, respectively (Figure 4C). While the empty vector control was larger in cell diameter its deformation resulted in a 0.8-fold decrease indicating that transduction directly affected the mechanical properties of the cells (*p* = 0.001). However, the CD97 knock down cells increased 1.7-fold in deformation (*p* < 0.00001) compared to the wildtype cells which could mainly be originated from the increase in size when considering the iso-elasticity lines (Figure 4C and S1).

Adhesion is important for HSPC and leukemic cell engraftment in the bone marrow niche. Therefore, we investigated the adhesion potential of CD97 knock down MV4-11 cells on a stromal layer *in vitro*. After one day of co-incubation 7 × 10^3^ cells carrying the empty vector were counted in the supernatant whereas 1.8 × 10^4^ of CD97 knock down cells were not attached (Figure 4D). This effect of the impaired adhesion was also significant after two days with 1.8 × 10^4^ vs. 2.9 × 10^4^ cells in the supernatant, but after three days it seemed to be saturated because only a slight difference was still detectable (Figure 4D).

## DISCUSSION

Leukemic and normal HSPCs reside within specialized niches in the bone marrow and interact via membrane proteins as well as soluble factors. Changes in these interactions alter hematopoietic stem cell fate, phenotype and behavior and may result in therapy resistance of leukemic cells. This is one reason for relapse and makes AML still difficult to treat. Thus, identification of markers to recognize and ultimately target leukemic cells is warranted. The combination of therapeutics and diagnostics called theragnostics offers the opportunity to generate an advanced molecular understanding of the disease, to develop more effective molecular targets and to design therapeutic agents based upon patient-specific biology the disease [17].

The EGF-TM7 molecule CD97 is poorly investigated in human normal as well as malignant HSPCs. In the murine system, CD97 expression was detected in hematopoietic stem cells of the bone marrow with highest levels in progenitor cells with the lowest colony forming potential [18]. The IL-8 mediated mobilization of HSPCs was inhibited by blocking anti-CD97 antibodies which confirms the function of CD97 expressing granulocytes in the bone marrow [19]. Moreover, higher granulopoietic activity was demonstrated in mice lacking either CD97 or the ligand CD55 [20].

To investigate a role of CD97 in malignant hematopoiesis, we initiated a comprehensive study in de novo AML and analyzed CD97 expression together with other cell surface molecules as well as mutation status. Interestingly, a higher CD97 expression (MFI ratio) in bone marrow blasts was associated with a lower percentage of CD34+ and/or CD117+ AML patients as well as presence of mutations in the *FLT3* and *NPM1* gene.

Bonardi and co-workers [21] identified CD97 as one of the plasma membrane associated proteins in two leukemic patient samples investigated by proteomics and transcriptome analyses. However, in contrast to our findings they allocated CD97 expression to the CD34+ group.

In another study, CD97 expression accounted for informative differences between normal and malignant cells [22] which are a prerequisite for the ability as diagnostic or therapeutic target. Primary cells derived from xenotransplanted patient samples were validated by flow cytometry at diagnosis and at early treatment time points. The comparison of changes in the fluorescence intensities revealed that although CD97 and CD58 expression did not appear to be modulated at all by chemotherapy, others were clearly reduced (CD102 and CD317) or increased (CD305 and CD63) in several cases [22].

Moreover, CD97 overexpression was detected in ALL subtypes which are defined by genetic abnormalities [23] which is in line with a high CD97 MFI ratio in 12 out of 16 predominantly common-B-ALL patients investigated in addition to our AML samples (data not shown).

These published data support the role of CD97 in the biology of leukemia but functional relations are still missing. Therefore, we focused our investigation on the association of increased CD97 expression and FLT3-ITD. Mutations of *FLT3* comprise one of the most frequently identified types of genetic alterations in AML. One-third of AML patients have mutations in this gene, and the majority involves internal tandem duplication in the juxtamembrane region of *FLT3*, leading to constitutive activation of downstream signaling pathways and aberrant cell growth [24].

The higher CD97 expression in *FLT3* mutated MV4-11 and MOLM-13 cells *in vitro* confirmed our findings from the patient samples. Leukemic cell lines with FLT3 wildtype expression like EOL-1 and HL-60, but also normal CD34+ HSPCs (not shown) displayed low CD97 expression levels. In contrast, OCI-AML3 cells which carry wildtype *FLT3* but mutated *NPM1* showed moderate CD97 levels which also correlated with our *in vivo* findings. Further evidence for a causative role of FLT3-ITD was found using different tyrosine kinase inhibitors. Sublethal doses of PKC412 or SU5614 which are able to block FLT3-ITD-mediated signaling resulted in decreased CD97 expression in MV4-11 and MOLM-13 but had no influence or an even reverse effect in the other cell lines. PKC412 (Midostaurin) is an inhibitor of tyrosine kinase, protein kinase C as well as VEGF and inhibits cell growth and phosphorylation of FLT3, STAT5, and ERK. It is a potent inhibitor of a spectrum of FLT3 activation loop mutations. SU5614 is a FLT3 inhibitor as well as a selective inhibitor of VEGF and PDGF receptor tyrosine kinases. Although these inhibitors have a rather broad spectrum regarding inhibition of phosphotyrosine kinases, a potential role of other tyrosine kinases in MV4-11 and MOLM-13 cells appears unlikely, because FLK1 protein (VEGFR2), the PDGFR (α and β isoform) receptor and the KIT protein were not expressed [25, 26]. Similar findings were observed for ATX expression which is linked to FLT3-ITD as well as the control of cellular proliferation and migration [26].

Next, we aimed at investigating effects of modulated CD97 expression on leukemic cellular behavior. Therefore, RNA interference experiments were performed in MV4-11 cells applying retroviral transduction. CD97 could be down-regulated to various extents by different shRNAs and this was correlated with the reduction of FLT3 expression confirming an association of these molecules *in vitro*. Moreover, induction of FLT3-ITD in normal CD34+ HSPC after retroviral transduction resulted in elevated CD97 expression levels (unpublished data).

Deregulation of CD97 expression has been implicated in the pathogenesis of different cancer entities, e.g. thyroid and colorectal carcinoma, most likely serving as a migration supporting molecule [14, 15, 27]. The interaction of leukemic cells with the bone marrow microenvironment requires regulation of surface molecules to mediate migration and adhesion processes. We hypothesized that CD97 acts as mediator of migration, cell deformability and adhesion. Indeed, CD97 knock down in FLT3-ITD expressing MV4-11 cells significantly inhibited the directed trans-well migration towards FCS which contains lysophosphatidic acid (LPA) as a major component. In prostate cancer cells, LPA receptor 1 (LPAR1) expression was sufficient to allow LPA-initiated migration and co-expression of CD97 and LPAR1 resulted in enhanced Rho-dependent invasion relative to LPAR1 alone [16]. Our data suggest that CD97 is linked to LPAR in FLT3-ITD positive leukemic cells and this association mediates the migration capacity of the cells which was significantly inhibited towards LPA after CD97 knock down. Moreover, CD97 mediated migration is at least in part Rho-dependent since pre-incubation with the Rho inhibitor CT04 resulted in a further decreased migration potential.

The majority of AML cells or cell lines expresses FLT3 as well as CXCR4 and migrates in response to SDF-1 [28]. RS4-11 acute leukemic cells, which co-express wildtype FLT3 and CXCR4, demonstrated synergistic migration to FLT3 ligand plus SDF-1. In our migration studies recombinant SDF-1 served as positive control and the attraction of MV4-11 cells was not affected by CD97 knock down suggesting that CD97 expression provides an additional pathway which mediates leukemic cell migration.

The migration capacity can be related to cell mechanics. Very recently, we introduced RT-DC, which allows on-the-fly analysis of deformed cell shape [29]. Using this technology, we provide evidence for decreased stiffness and therefore higher deformability of CD97 knock down cells compared to those carrying the empty vector or the wildtype, which could mainly be originated from an increase in cell size. It is conceivable that the reduced FLT3 expression due to CD97 knock down could lead to reduced Ezrin activation as a consequence in reduced cell rigidity. Faure et al. described that reduced Ezrin activation resulted in disanchoring of the cortical actin cytoskeleton from the plasma membrane and decreased cellular rigidity [30]. Mechanical phenotype means that something controlling cell stiffness inside the cell is different. This is likely the cytoskeleton, probably amongst others less investigated factors such as general molecular crowding, poroelasticity or condensation of supramolecular complexes. So, an altered mechanical phenotype is a clear marker for intracellular changes that are worth to be explored in future due to their likely connection to some aspects of cell function.

Adhesion to the stromal niche is crucial for leukemic (stem) cells because it directly supports self-renewal, proliferation and arrest of differentiation and protects from damage by chemotherapy or kinase inhibitors [31]. A few molecules and pathways involved in these processes are described so far. The transmembrane glycoprotein CD44 modulates interactions of leukemic stem cells with hyaluronan; ECM components, including heparin sulphate; and a range of growth factor ligands to promote CD44/ligand/RTK complex formation and signal transduction [31]. Manipulation of CD44 function with the H90 monoclonal antibody resulted in marked reduction of the leukemic burden in NOD/SCID mice transplanted with primary AML cells through alteration of cell fate and abrogation of leukemic cell homing [32]. We could demonstrate that CD97 knock down in MV4-11cells resulted in an impaired adhesion on a MSC monolayer. Binding partners of CD97 as integrins α5β1 and αvβ3 [13] and CD90 (Thy-1) [12] are expressed on MSCs or in the case of the glycosaminoglycan chondroitin sulfate as component of the extracellular matrix [33]. Further investigations will provide detailed information about the exact pathways mediating the observed effects *in vitro*.

In summary, we provide first evidence for a potential functional relevance of CD97 in FLT3-ITD AML. Future studies will have to confirm its role as a new diagnostic and therapeutic target in AML.

## MATERIALS AND METHODS

### Patient samples

All patients were part of the prospective DSIL AML96 protocol [16] and had provided written consent in accordance with the Declaration of Helsinki. The study had been approved by the ethical board of the Technische Universität Dresden.

We studied 385 bone marrow samples from patients with de novo acute leukemia, comprising AML M0-2 (*n* = 272), AML M3 (*n* = 16), AML M4/5 (*n* = 91) and AML M6/7 (*n* = 6). FLT3-ITD mutations and NPM1-mutations were detected as reported in detail previously [6, 17]. MDS samples (*n* = 15) as well as normal bone marrow of healthy donors (*n* = 10) were included as controls. CD34+ HSPCs were purified from leukapheresis samples using CD34 antibody conjugated magnetic beads according to the manufacturer's instructions (Miltenyi Biotec, Bergisch Gladbach, Germany). The purity was >95% as assessed by flow cytometry, and the viability as measured by trypan blue exclusion was >96%.

### Cell lines

Leukemic MV4-11, MOLM-13, OCI-AML3, EOL-1 and HL-60 cell lines were purchased from the German Collection of Microorgansims and Cell Cultures (DSMZ, Braunschweig, Germany) and cultured in RPMI or DMEM (OCI-AML3) medium supplemented with 10% fetal calf serum (FCS). Treatment with protein kinase inhibitors PKC412 (Midostaurin) and SU5614 (both from Sigma-Aldrich, Taufkirchen, Germany) was performed for 12 hours at a sublethal concentration of 0.5 μM and 0.1 μM, respectively.

### Lentiviral transfection of short hairpin RNAs

To produce lentiviral vector particles, HEK293T cells were transfected with a plko1.6 vector containing a short hairpin RNA (shRNA) against CD97 (clones: TRCN0000008234, TRCN0000008235, TRCN0000008236, TRCN0000008237, TRCN0000008238, Sigma-Aldrich) in combination with the packaging plasmids psPAX and pVSVg using PEI [18]. Lentivirus-vector containing media were collected 48 h after transfection. MV4-11 cells were infected with lentiviral vector particles (0.5 x viral supernatant) in the presence of 1 mg/mL protamine. Selection of transduced cells was carried out with puromycin. Efficient CD97 knock down was evaluated by flow cytometry.

### Flow cytometry

The AML patient samples were analyzed by a standardized routine 8-color immunophenotypic measurement on a FACS Canto II as described recently [19]. For investigation of CD97 expression a 4-color immunophenotypic measurement was performed using the following antibodies: CD34 PerCp5.5, CD117 FITC, CD97 APC, CD45 V500. Thus, CD97 expression was analyzed as a ratio of mean fluorescence intensity (MFI) of blasts, granulocytes, or monocytes in relation to the MFI of lymphocytes in each sample. In cell lines, CD97 was stained by the CD97 APC (clone MEM-180, AbD Serotec, Puchheim, Germany), FLT3 by CD135 APC (eBioscience, Frankfurt, Germany) and measured at a FACS Calibur.

### Proliferation

MV4-11 cells transduced with empty vector or CD97 shRNA (1 × 10^4^/well in a 96-well plate) were cultured for 1 day, and an MTT assay was performed according to the protocol (Roche Diagnostics GmbH, Mannheim, Germany). The absorbance was measured at 570 and 650 nm. Medium was used as a negative control.

### Apoptosis

Detection and discrimination of apoptotic, necrotic and dead cells were performed using an Annexin V-FITC kit (Miltenyi Biotec) following the manufacturer's instructions.

### Trans-well migration

The migration potential of MV4-11 cells was tested by using 8 μm pore size Costar Trans-wells (Corning, Cambridge, MA, USA). 1 × 10^5^ cells were washed, re-suspended in 100 μl RPMI medium supplemented with 0.1% bovine serum albumin (BSA) and loaded in the upper chamber. 500 μl medium containing 10% FCS, recombinant human SDF-1 (100 ng/ml, PeproTech, Hamburg, Germany) or serially diluted lysophosphatidic acid (LPA, Sigma-Aldrich) were placed into the lower well. After incubation for 5 h at 37°C, the trans-migrated cells were counted by flow cytometry for 30 sec. A sample of non-migrated cells served as reference.

### Real-time deformability cytometry (RT-DC)

MV4-11 cells were centrifuged at 115 g for 5 min and re-suspended in a solution of phosphate buffered saline (PBS) and 0.5% (w/v) methylcellulose (Sigma-Aldrich) to a final concentration of 1 × 10^6^ cells/ml. A final sample volume of 100 μl was used for analysis. The cell suspensions were kept at 37°C before being drawn into a 1 ml-syringe and mechanically characterized using RT-DC as described previously [20]. Briefly, cells are being flushed through the 20 μm constriction of a microfluidic chip made of polydimethylsiloxane where they are being deformed due to hydrodynamic interactions. At a constant flow rate of 0.04 μl/s cell deformation d and size is determined in real-time and quantified to:

$$D=1-c=1-\frac{2\sqrt{\pi A}}{l}$$

where c is the circularity of a cell, A the projected surface area l and its perimeter. Since deformation and size are not independent quantities the mechanical properties of the cells are originated from an analytical model and described by iso-elasticity lines (curved lines in Figure 4C) [20].

### Statistics

Data were analyzed using the GraphPad Prism statistical PC program (GraphPad, San Diego, CA, USA). Results from at least three independent experiments are presented as the mean ± standard error of the mean (SEM, presented as error bars). Statistical comparisons were performed using either one-way or two-way ANOVA with Bonferroni's post-test.

For real-time deformability cytometry statistical significance was obtained from a one sample *t*-test after bootstrapping the data sets to be compared. Bootstrapping is required since the projected deformation data is skewed and is carried out for *n* = 100,000 iterations.

## SUPPLEMENTARY FIGURE


