# Supplementary material for: Association of the EGF-TM7 receptor CD97 expression with FLT3-ITD in acute myeloid leukemia
Source: Oncotarget. 2015 Oct 7;6(36):38804–15. doi: 10.18632/oncotarget.5661 (PMC4770738; doi:10.18632/oncotarget.5661)
Supplement: Supplementary file 1 [file oncotarget-06-38804-s001.pdf]

SUPPLEMENTARY FIGURE

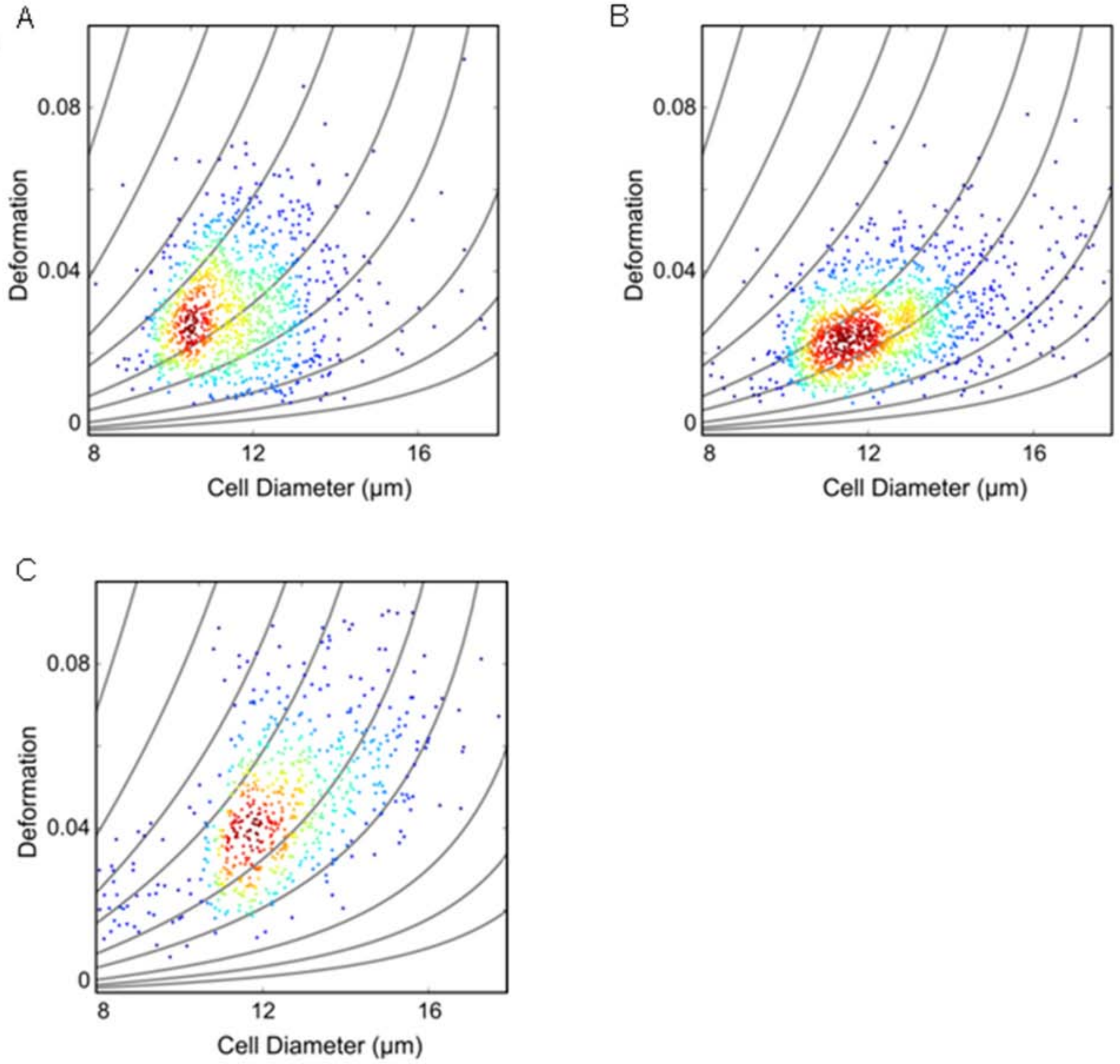

Supplementary Figure S1: RT-DC on MV-11 cells. Mechanical properties of **A.** wildtype, **B.** empty vector and **C.** down-regulated MV-11 cells each carried out in a separate experiment at a flow rate of 0.04 µl/s in a 20 µm × 20 µm channel.
